# Assessing performance and stability of yellow rust resistance, heat tolerance, and agronomic performance in diverse bread wheat genotypes for enhancing resilience to climate change under Egyptian conditions

**DOI:** 10.3389/fpls.2022.1014824

**Published:** 2022-11-10

**Authors:** Eman M. A. Megahed, Hassan A. Awaad, Ismail E. Ramadan, Mohamed I. E. Abdul-Hamid, Abdallah A. Sweelam, Doaa R. El-Naggar, Elsayed Mansour

**Affiliations:** ^1^ Wheat Research Department, Field Crops Research Institute, Agricultural Research Center, Giza, Egypt; ^2^ Department of Crop Science, Faculty of Agriculture, Zagazig University, Zagazig, Egypt; ^3^ Wheat Disease Department, Plant Pathology Research Institute, Agricultural Research Center, Giza, Egypt

**Keywords:** final rust severity, average coefficient of infection, area under disease progress curve, heat tolerance indices, yield traits, cluster analysis, principal component analysis, heatmap and hierarchical clustering

## Abstract

Yellow rust and heat stress adversatively impact the growth and production of bread wheat in particular under rising adverse environmental conditions. Stability of grain yield is a pivotal purpose of plant breeders to improve wheat production and ensure global food security especially under abrupt climate change. The objective of this study was to assess the performance and stability of diverse bread wheat genotypes for yellow rust resistance, heat stress, and yield traits. The studied genotypes were evaluated in two different locations under two sowing dates (timely and late sowing) during two growing seasons. The obtained results displayed significant differences among the tested locations, sowing dates, and genotypes for most measured traits. The yellow rust measurements evaluated under the field conditions including final rust severity (FRS), the average coefficient of infection (ACI), and area under disease progress curve (AUDPC) revealed that Giza-171, Misr-1, Gemmeiza-12, Shandweel-1, Sids-13, Line-1, Line-2, and Line-55 had better resistance. Based on heat sensitivity measurements, Line-1 and Line-2 followed by Line-35, Shandweel-1 and Line-55 were classified as more tolerant to heat stress compared with the remaining genotypes. The genotypes Line-55, Gemmeiza-12, Giza-171, Line-1, Line-2, and Misr-1 were able to maintain acceptable agronomic performance under timely and late sowing dates in all evaluated environments. Different statistical procedures were employed to explore the adaptability and stability of tested genotypes *i.e.*, joint regression, stratified ranking, Wricke's Ecovalence values, cultivar superiority, additive main effects, and multiplicative interaction (AMMI), AMMI stability value, and genotype plus genotype-by-environment interaction (GGE). The applied stability parameters were quite similar for describing the stability of the evaluated wheat genotypes. The results indicated that Gemmeiza-12, Giza-171, Sids-12, Sids-13, Misr-1 Shandweel-1, Line-1, Line-2, and Line-55 were desirable and stable. The heatmap and hierarchical clustering were exploited for dividing the evaluated bread wheat genotypes into different clusters based on yellow rust resistance measurements, heat tolerance indices, and agronomic performance. Line-1 and Line-2 had the best performance for all rust resistance, heat tolerance, and agronomic performance followed by Giza-171, Line-55, Line-35, Gemmeiza-12, Shandweel-1, Misr-1, and Sids-13. In conclusion, our findings provide evidence of utilizing promising genotypes in rust resistance, heat tolerance, and agronomic performance in breeding programs for improving wheat grain yield stability mainly under climate change.

## Introduction

Wheat (*Triticum aestivum* L.) is an irreplaceable cereal crop and a vital source of carbohydrates (Shewry and Hey, 2015). Its global cultivated area is approximately 219 million hectares, which produces around 761 million tons yearly (FAOSTAT, 2022). The total production of wheat in Egypt is about 9 million tons while the consumption is around 20 million tons. Egypt is considered one of the greatest importers worldwide with 10 million tons annually (FAOSTAT, 2022). Furthermore, the gap between exaggerated consumption and wheat production is growing due to population growth and the negative effects of climate change on wheat production. Consequently, improving wheat production has become a decisive requirement to cope with current restrictions for narrowing the gap between production and consumption to ensure global food security.

The biotic and abiotic stresses including yellow rust and heat stress cause adverse impacts on bread wheat production, particularly under abrupt climate change (Kamara et al., 2021; Martínez-Moreno et al., 2022). Yellow rust caused by *Puccinia striiformis* f.sp*. tritici* is the most common wheat disease that triggers considerable destruction in many regions worldwide, accordingly, threatening its production (Chen, 2005; Nsabiyera et al., 2018). Genetic resistance to yellow rust is the most economical and environmentally approach to control this disease with no additional costs (Chen, 2007; Feng et al., 2018). The recent climatological extremes including high temperature are projected to deleteriously impact wheat growth and productivity. Terminal heat stress at the post-heading stage causes considerable yield reduction due to stress at a critical stage, *i.e*., anthesis and grain filling (Marcela et al., 2017). At flowering, it causes destructive impacts on pollen fertility and seed setting which lead to low grain number per spike (Talukder et al., 2014; Ullah et al., 2022). Moreover, during the grain filling period, it shortens the period of grain filling and diminishes grain weight (Djanaguiraman et al., 2020). Accordingly, it is irreplaceable to improve the heat tolerance of wheat genotypes.

The environmental factors considerably impact rust infection. The rust development is significantly impacted by minimum temperature, maximum relative humidity, and sowing date (Naseri and Marefat, 2019). Early sowing has considerable importance to avoid rust severity, while severe infection occurs in late sowing (Duveiller et al., 2007; Naseri and Kazemi, 2020). Moreover, late sowing postpones the filling stage and results in an increase in the temperature during the filling stage (Wang et al., 2018). In addition, late sowing significantly reduces the duration of grain filling which was reported in earlier reports demonstrating that late sowing causes a considerable reduction in the grain filling period (Chauhan et al., 2009; Farooq et al., 2011; Tiwari et al., 2012; Elbasyoni, 2018; Wang et al., 2018). Hence, late sowing induces heat stress, especially during the grain filling period, accordingly differentiating sensitive and tolerant wheat genotypes.

Variation in the climate and soil conditions leads to significant alterations in wheat production. Thus genotype × environment interaction is a decisive issue facing wheat breeders (Mohammadi et al., 2012). In the multi-location field experiment, a significant G×E interaction diminishes the association between genotypic and phenotypic values as well as the progress from the selection procedure. Accordingly, plant breeders frequently evaluate the genotypes across various environments to explore their adaptability and stability (Erkul et al., 2010; Mansour et al., 2018b). Recently, great challenges are faced by wheat breeders and producers to increase grain yield without sacrificing stability under the prevailing climatic changes (Sandro et al., 2022).

Different statistical procedures are used to explore the stability of assessed genotypes such as Additive main effects and multiplicative interaction (AMMI) (Gauch, 1992), joint regression (Eberhart and Russell, 1966), Wricke’s Ecovalence (Wricke, 1962), and Cultivar superiority (Lin and Binns, 1988). Several researchers assessed the phenotypic stability of yield performance using different statistical procedures and deduced different stability degrees across multi-environment trials (Mansour et al., 2018a; Alipour et al., 2021; Awaad, 2021; Omrani et al., 2022). Although several studies assessed yellow rust resistance, heat tolerance, agronomic performance, and yield stability of wheat genotypes severally, additional investigation is needed to assess these biotic and abiotic stresses alongside the agronomic performance. Hence, the objective of this study was to evaluate rust resistance, heat resilience, and yield potentiality, as well as characterize the adaptability and stability of diverse bread wheat genotypes under a Mediterranean environment employing several statistical procedures.

## Materials and methods

### Plant materials and experimental sites

Twelve bread wheat (*Triticum aestivum* L.) genotypes were utilized in the current study. The pedigree and origin of the evaluated genotypes are shown in **Table S1**. The studied genotypes were selected based on their different performance in previous trials. Eight field trials were conducted at two diverse locations in Egypt (**Table 1**); Kafer El-Hamam, Sharqia (31°51’ N and 31°61’ E) and Sakha, Kafr El-Sheikh (31°08’ N, 30°94’ E) during two growing seasons (2015-16 and 2016-17) at two sowing dates (20 November and 15 December). Meteorological data of the experimental sites maintained by the Egyptian Meteorological Authority (EMA) are shown in **Table S2**. The two locations’ soil is clay but Kafer El Hamam is richer in available nitrogen and potassium while vice versa in available phosphorous (**Table S3**).

**Table 1:** Description of the performed field trails.

Location	Sowing date	Code	Grain yield (t/ha)	Days to heading	Days to maturity
Kafer El-Hamam, Sharqia	20 November 2015	E1	6.09	101.31	154.49
Kafer El-Hamam, Sharqia	15 December 2015	E2	4.47	98.04	135.91
Sakha, Kafer El-Sheikh	20 November 2015	E3	6.39	99.08	154.19
Sakha, Kafer El-Sheikh	15 December 2015	E4	4.21	91.06	132.81
Kafer El-Hamam, Sharqia	20 November 2016	E5	6.44	91.02	138.33
Kafer El-Hamam, Sharqia	15 December 2016	E6	4.01	87.08	126.00
Sakha, Kafer El-Sheikh	20 November 2016	E7	6.56	89.53	138.47
Sakha, Kafer El-Sheikh	15 December 2016	E8	4.45	86.42	122.59

The experimental materials were laid out in a randomized complete block design with three replications. The seeds of the studied wheat genotypes were sown by hand drilling in plots containing six rows of 3 meters long with 20 cm apart. Surface irrigation was applied at both locations as the standard systems used at both sites. The total amount of water of approximately 4000 m^3^ ha^–1^ distributed across each season. The tested genotypes were sown 25 days after the normal sowing date (15 December) to expose the plant genotypes to an appropriate environment for rust development, disease epidemiology, and heat stress during the grain filling period as obvious in **Table S2**. The recommended agricultural practices were followed as usual for wheat in each location.

### Measured traits

#### Yellow rust measurements

The field trials were surrounded by a square meter border of the rust susceptible cultivar (Morocco) to act as a spreader. Following the procedure adopted by Tervet and Cassel (1951) the spreader received an additional artificial inoculation of the pathogen at the booting stage. The used rust races were 106E166, 128E28, 159E255 and 450E214. The inocula (urediniospores mixture) were obtained from the yellow rust greenhouse in Wheat Diseases Research Department, Plant Pathology Research Institute, Egypt, and mixed with talcum powder at the rate of 1:20 (w.w). Rust measurements were recorded three times, the first score was recorded when rust symptoms have fully developed in comparison with the susceptible cultivar (Morocco). The second and the third scores were recorded at intervals of one week. Final rust severity (FRS), the average coefficient of infection (ACI), and the area under the disease progress curve (AUDPC) were measured. FRS was determined as a percentage (%) of leaf area covered by yellow rust pustules following the modified Cobb’s scale of 0-100% according to Peterson et al. (1948) and Singh et al. (2008) alongside the infection type. The small pustules surrounded by necrosis were characterized as resistant type (R), small to medium pustules surrounded by green island as moderately resistant (MR), medium-sized pustules without necrosis or chlorosis as moderately susceptible (MS), large-sized pustules without chlorosis or necrosis as susceptible (S) (Stubbs et al., 1986). ACI was determined according to the formula of Peterson et al. (1948), yellow rust severity × infection type. Infection type based on resistant (R)=0.2, moderately resistant (MR)=0.4, moderately susceptible (MS) =0.8, and susceptible (S) =1, as described by Stubbs et al. (1986). AUDPC was assessed according to the formula adopted by Pandey et al. (1989), AUDPC = D [½ (Y1+Yk) +Y2+Y3 + …. +Yk-1], where D is the days between consecutive recordings (time intervals), Y1+ Yk is the sum of the first and the last disease scores and Y2 + Y3 + …. + Yk-1 is the sum of all in between disease scores.

#### Heat stress measurements

The tested wheat genotypes were evaluated for heat stress under timely sowing as control and late sowing to expose the plants to heat stress at the anthesis and grain-filling period. Heat stress tolerance measurements were assessed as follows: yield reduction ratio (YRR)=1-(Ῡs/Ῡp) following Golestani Araghi and Assad (1998), stress sensitivity index (SSI)=[1-(Ys/Yp)]/[1-(Ῡs/Ῡb)] as outlined by Fischer and Wood (1979), stress tolerance index (STI)=(Ys×Yp)/(Ῡp)^2^ as presented by Fernandez (1992) and relative performance (P)=(Ys/YP)/(Ῡs/Ῡp) according to Hossain et al. (1990). Where, Yp, Ys, Ῡp, and Ῡs refer to yield under normal conditions, yield under stress, mean yield under normal, and mean yield under stress conditions, respectively.

### Yield traits

Number of grains per spike was determined from ten spikes collected randomly from each plot. Thousand-grain weight was measured as the weight of 1000-grains was sampled from each plot. Grain yield was measured as the weight of grain harvested per four middle rows and converted to tons per hectare.

### Statistical analysis

Combined analysis of variance over environments were applied to determine the effects of the environment, genotype, and G×E interaction. Stability parameters were calculated according to the four statistical procedures. Regression coefficient (b_i_) and deviation mean square from linear regression ( ${\text{S}}_{d}^{2}$ ) were calculated as described by Eberhart and Russell (1966). The stratified ranking technique of Fox et al. (1990) was employed to classify the genotypes. The stratified ranking of each genotype was stated as the proportion of environments where that genotype ranked in all entries (TOP). Wricke’s Ecovalence (Wricke, 1962), cultivar superiority of Lin and Binns (1988), AMMI analysis (Gauch, 2006), and AMMI’s stability value (ASV) disclosed by Purchase (1997) were performed using Genstat (version 19). The Heatmap, hierarchical clustering, and principal component analyses were applied using R statistical software (version 4.1.1).

## Results

### Analysis of variance

The combined analysis of variance displayed significant effects for genotypes (G), environments (E), and their interaction (G×E) (**Table 2**). The evaluated genotypes exhibited the largest proportion of sums of squares of yellow rust measurements; rust severity, the average coefficient of infection, and area under disease progress curve. Similarly, the genotypic effect exhibited the highest contribution of the total variation for heat tolerance indices, yield reduction ratio, stress sensitivity index, stress tolerance index, and relative performance. On the other hand, the environmental effect displayed the highest proportion of the total variation of yield traits; number of grains/spike, 1000-grain weight, and grain yield followed by genotype by environment and genotypic effects. The environmental variation in yield traits was obviously dominated by the sowing date effect. The genotype by environment interaction (G×E) was significant in all measured traits. The two-way and three-way interactions among location, sowing date, and genotype were significant for most traits.

**Table 2:** Analysis of variance for the evaluated parameters of twelve bread wheat genotypes tested in two locations (Kafer El-Hamam and Sakha) during two growing seasons in 2015-2016 and 2016-2017 under timely and late sowing dates.

Sources ofvariation	df	Rust severity			Average coefficient of infection			Area under disease progress curve			No. of grains/spike			1000-grain weight			Grain yield		
		MS		%SS	MS		%SS	MS		%SS	MS		%SS	MS		%SS	MS		%SS
Environment (E)	7	734.5	**	3.00	952.2	**	2.96	16866	**	2.028	869.6	**	60.54	846.5	**	39.10	46.20	**	60.45
Season (S)	1	136.1		0.08	283.6		0.13	17191		0.30	0.20		0.01	382.2	**	2.52	0.42		0.08
Location (L)	1	153.1		0.09	423.9		0.19	9825		0.17	30.68	*	0.31	382.2	**	2.52	1.63	*	0.30
Sowing date (D)	1	3308	**	1.93	4495	**	1.99	51224	**	0.88	5825	**	57.93	4897	**	32.32	313.8	**	58.65
S × L	1	168.1		0.1	129.9		0.06	2333		0.04	16.53		0.16	219.8	**	1.45	1.25	*	0.23
S × D	1	5.01		0.05	215.3		0.1	18248		0.31	0.15		0.00	9.60		0.06	2.45	**	0.46
L × D	1	2.30		0.06	1.20		0.05	1824		0.03	141.4	**	1.41	29.28	**	0.19	0.28		0.05
S×L×D	1	1369	**	0.8	1116	*	0.5	17414		0.30	73.61	**	0.73	4.71		0.03	3.64	**	0.68
Genotype (G)	11	11289	**	72.37	13843	**	67.53	352666	**	66.63	98.93	**	10.82	474.2	**	34.42	5.20	**	10.68
G × E	77	308.1	**	13.83	356.4	**	12.17	10350	**	13.69	22.98	**	17.60	42.35	**	21.52	1.23	**	17.70
S × G	11	285.3	**	1.83	489.9	**	2.39	8225		1.55	12.86	*	1.41	138.6	**	10.06	1.19	**	2.45
L × G	11	446.6	**	2.86	724.4	**	3.53	19359	**	3.66	43.76	**	4.79	17.67	**	1.28	1.47	**	3.01
D×G	11	337.0	**	2.16	472.7	*	2.31	17693	**	3.34	46.84	**	5.12	37.34	**	2.71	3.04	**	6.25
S×L×G	11	590.1	**	3.78	354.6		1.73	14010	**	2.65	9.77		1.07	12.46	**	0.90	0.60	*	1.24
S×D×G	11	133.9		0.86	187.0		0.91	7017		1.33	17.09	**	1.87	73.94	**	5.37	0.46		0.95
L×S×G	11	126.0		0.81	61.1		0.3	1386		0.26	17.52	**	1.92	6.64		0.48	0.84	**	1.73
S×L×D×G	11	237.7	**	1.52	205.4		1.0	4763		0.90	13.02	*	1.42	9.77	**	0.71	1.01	**	2.08
Residual	190	93.9		10.4	204.4		17.22	5261		17.17	5.80		10.97	3.90		4.89	0.31		11.08
Total	287	597.9			785.7			20287			35.03			52.80			1.86		
**Sources of variation**	**df**	**Yield reduction ratio**			**Stress sensitivity Index**			**Stress tolerance index**			**Relative performance**								
		MS		%SS	MS		%SS	MS		%SS	MS		%SS						
Environment (E)	3	0.071	**	5.99	0.01		0.10	0.07	**	5.99	0.002		0.15						
Season (S)	1	0.116	**	3.26	0.003		0.01	0.12	**	3.26	0.001		0.05						
Location (L)	1	0.018		0.51	0.02		0.05	0.02		0.51	0.005		0.06						
S×L	1	0.079	**	2.23	0.01		0.04	0.08	**	2.23	0.01		0.08						
Genotype (G)	11	0.116	**	36.1	1.10	**	37.05	0.12	**	36.1	0.26	**	38.61						
G×E	33	0.035	**	32.31	0.34	**	34.26	0.03	**	32.31	0.08	**	34.74						
S×G	11	0.022	*	6.92	0.26	**	8.78	0.02	*	6.92	0.05	*	6.86						
L×G	11	0.042	**	13.04	0.41	**	13.59	0.04	**	13.04	0.10	**	13.99						
S×L×G	11	0.040	**	12.35	0.35	**	11.9	0.04	**	12.35	0.09	**	13.89						
Residual	94	0.010		25.18	0.1		28.06	0.01		25.18	0.02		26.10						
Total	143	0.025			0.23			0.02			0.05								

%SS: percentages of sums of squares, *P< 0.05, **P< 0.01.

### Resistance to yellow rust

Final rust severity (FRS), the average coefficient of infection (ACI), and area under disease progress curve (AUDPC) were employed to determine the variability among wheat genotypes for durable resistance. Highly significant differences (P< 0.01) were detected among genotypes indicating the presence of genetic variances. The results displayed that Giza-171, Misr-1, Gemmeiza-12, Sids-13, Shandweel-1, Line-1, Line-2, and Line-55 recorded the lowest rust severity (**Table 3**). The remaining genotypes exhibited higher rust severity particularly Sids-12, Sakha-93, Line-3, and Line-35. Generally, late sowing (E2, E4, E6, and E8) displayed higher rust severity compared to timely sowing (E1, E3, E5, and E7). The tested genotypes behaved the same trend for ACI and AUDPC parameters as FRS (**Tables 4**, **5**). Line-55, Giza-171, Misr-1, Gemmeiza-12, Sids-13, Shandweel-1, and Line-2 recorded the lowest ACI and AUDPC values under all evaluated environments. Otherwise, the highest values of ACI and AUDPC were registered by Sids-12, Sakha-93, Line-35, and Line-3 under timely and late sowing. Heat stress tolerance

**Table 3:** Final rust severity (FRS) of the evaluated twelve bread wheat genotypes in two locations (Kafer El-Hamam and Sakha) during two growing seasons in 2015-2016 and 2016-2017 under timely and late sowing dates.

Genotype	— E1 —		— E2 —		— E3—		— E4 —		— E5 —		— E6 —		— E7 —		— E8 —	
	DS%	IT	DS%	IT	DS%	IT	DS%	IT	DS%	IT	DS%	IT	DS%	IT	DS%	IT
Giza-171	3 ^c^	R	3 ^b^	S	5 ^c^	MS	10 ^c^	MS	0 ^b^	0	0 ^d^	0	3 ^c^	R	3 ^c^	R
Misr-1	0 ^c^	0	3 ^b^	0	0 ^c^	0	3 ^c^	S	5 ^b^	MS	5 ^d^	MR	3 ^c^	S	10 ^c^	S
Gemmeiza-12	3 ^c^	R	3 ^b^	S	10 ^c^	S	10 ^c^	S	3 ^b^	R	5 ^d^	S	3 ^c^	S	10 ^c^	S
Sids-12	30 ^b^	S	70 ^a^	S	70 ^a^	S	70 ^a^	S	60 ^a^	S	70 ^a^	S	50 ^a^	S	70 ^a^	S
Sids-13	3 ^c^	R	3 ^b^	R	10 ^c^	S	10 ^c^	S	3 ^b^	R	10 ^cd^	MS	10 ^bc^	S	10 ^c^	S
Shandweel-1	0 ^c^	0	3 ^b^	MS	3 ^c^	S	5 ^c^	S	3 ^b^	S	5 ^d^	S	3 ^c^	S	10 ^c^	S
Sakha-93	50 ^a^	S	70 ^a^	S	70 ^a^	S	70 ^a^	S	50 ^a^	S	70 ^a^	S	60 ^a^	S	70 ^a^	S
Line-35	20 ^b^	S	60 ^a^	S	30 ^b^	S	50 ^b^	S	10 ^b^	S	20 ^bc^	S	20 ^b^	S	60 ^a^	S
Line-1	0 ^c^	0	5 ^b^	MS	5 ^c^	MR	5 ^c^	S	0 ^b^	0	30 ^b^	S	20 ^b^	S	30 ^b^	S
Line-2	3 ^c^	MS	3 ^b^	MS	0 ^c^	0	10 ^c^	R	10 ^b^	R	10 ^cd^	R	0 ^c^	0	10 ^c^	MR
Line-3	50 ^a^	S	60 ^a^	S	10 ^c^	S	10 ^c^	S	50 ^a^	S	60 ^a^	S	20 ^b^	S	60 ^a^	S
Line-55	0 ^c^	0	0 ^b^	0	0 ^c^	0	5 ^c^	MR	0 ^b^	0	0 ^d^	0	0 ^c^	0	0 ^c^	0

E1-E8 are the evaluated environments as presented in **Table 1**.

DS%: rust severity as a percentage of leaf area covered by yellow rust pustules following the modified Cobb’s scale of 0-100%.

IT, infection type; 0, No infection detected, R, Resistant small pustules surrounded by necrosis, MR, Moderately resistant; small to medium pustules surrounded by green island, MS, Moderately susceptible; medium-sized pustules without necrosis or chlorosis, S, susceptible large sized pustules without chlorosis or necrosis. The same letters under the same environment are not significantly different by the least significant difference at p ≤ 0.05.

**Table 4:** Average coefficient of infection (ACI) of the evaluated twelve bread wheat genotypes in two locations (Kafer El-Hamam and Sakha) during two growing seasons in 2015-2016 and 2016-2017 under timely and late sowing dates.

Genotype	E1	E2	E3	E4	E5	E6	E7	E8
Giza-171	0.6 ± 0.02 ^c^	3.0 ± 0.17 ^b^	4.0 ± 0.85 ^c^	8.0 ± 0.67 ^c^	0.0 ± 0.00 ^b^	0.0 ± 0.00 ^c^	0.6 ± 0.05 ^b^	0.6 ± 0.04 ^c^
Misr-1	0.0 ± 0.00 ^c^	1.2 ± 0.08 ^b^	0.0 ± 0.00 ^c^	3.0 ± 0.28 ^c^	4.0 ± 1.67 ^b^	2.0 ± 0.54 ^c^	3.0 ± 0.57 ^b^	10.0 ± 1.11 ^c^
Gemmeiza-12	0.6 ± 0.04 ^c^	3.0 ± 0.18 ^b^	10.0 ± 0.97 ^c^	10.0 ± 1.54 ^c^	0.6 ± 0.04 ^b^	5.0 ± 1.21 ^c^	3.0 ± 0.81 ^b^	10.0 ± 1.23 ^c^
Sids-12	30.0 ± 1.55 ^b^	70.0 ± 2.64 ^a^	70.0 ± 2.75 ^a^	70.0 ± 2.53 ^a^	60.0 ± 2.18 ^a^	70.0 ± 3.25 ^a^	50.0 ± 2.82 ^a^	70.0 ± 2.82 ^a^
Sids-13	0.6 ± 0.07 ^c^	0.6 ± 0.03 ^b^	10.0 ± 1.33 ^c^	10.0 ± 0.00 ^c^	0.6 ± 0.03 ^b^	8.0 ± 1.21 ^c^	10.0 ± 1.29 ^b^	10.0 ± 1.29 ^c^
Shandweel-1	0.0 ± 0.00 ^c^	2.4 ± 0.22 ^b^	3.0 ± 0.52 ^c^	5.0 ± 0.95 ^c^	3.0 ± 0.84 ^b^	5.0 ± 0.87 ^c^	3.0 ± 0.52 ^b^	10.0 ± 1.53 ^c^
Sakha-93	50.0 ± 2.75 ^a^	70.0 ± 2.64 ^a^	70.0 ± 2.88 ^a^	70.0 ± 1.53 ^a^	50.0 ± 2.35 ^a^	70.0 ± 3.28 ^a^	60.0 ± 2.31 ^a^	70.0 ± 2.27 ^a^
Line-35	20.0 ± 0.00 ^b^	60.0 ± 1.55 ^a^	30.0 ± 1.77 ^b^	50.0 ± 2.65 ^b^	10.0 ± 1.02 ^b^	20.0 ± 0.00 ^b^	20.0 ± 1.64 ^b^	60.0 ± 1.84 ^a^
Line-1	0.0 ± 0.00 ^c^	4.0 ± 0.85 ^b^	2.0 ± 0.19 ^c^	5.0 ± 0.55 ^c^	0.0 ± 0.00 ^b^	30.0 ± 1.38 ^b^	20.0 ± 1.13 ^b^	30.0 ± 0.77 ^b^
Line-2	2.4 ± 0.18 ^c^	2.4 ± 0.19 ^b^	0.0 ± 0.00 ^c^	2.0 ± 0.21 ^c^	2.0 ± 0.54 ^b^	2.0 ± 0.51 ^c^	0.0 ± 0.00 ^b^	4.0 ± 0.89 ^c^
Line-3	50.0 ± 2.54 ^a^	60.0 ± 1.55 ^a^	10.0 ± 1.23 ^c^	10.0 ± 1.14 ^c^	50.0 ± 1.65 ^a^	60.0 ± 2.38 ^a^	20.0 ± 0.00 ^b^	60.0 ± 2.82 ^a^
Line-55	0.0 ± 0.00 ^c^	0.0 ± 0.00 ^b^	0.0 ± 0.00 ^c^	2.0 ± 0.15 ^c^	0.0 ± 0.00 ^b^	0.0 ± 0.00 ^c^	0.0 ± 0.00 ^b^	0.0 ± 0.00 ^c^

E1-E8 are the evaluated environments as presented in **Table 1**. The same letters under the same environment are not significantly different by the least significant difference at p ≤ 0.05.

**Table 5:** The area under disease progress curve (AUDPC) of the evaluated twelve bread wheat genotypes in two locations (Kafer El-Hamam and Sakha) during two growing seasons in 2015-2016 and 2016-2017 under timely and late sowing dates.

Genotype	E1	E2	E3	E4	E5	E6	E7	E8
Giza-171	0.00 ± 0.00 ^e^	18.90 ± 1.60 ^e^	22.402.01± ^b^	17.73 ± 1.43 ^c^	0.00 ± 0.00 ^c^	0.00 ± 0.00 ^e^	0.70 ± 0.06 ^c^	0.70 ± 0.03 ^e^
Misr-1	1.87 ± 0.07 ^e^	0.00 ± 0.00 ^e^	0.00 ± 0.00 ^b^	20.53 ± 1.39 ^c^	14.47 ± 1.05 ^c^	5.13 ± 0.35 ^e^	9.10 ± 0.67 ^c^	9.10 ± 0.59 ^e^
Gemmeiza-12	0.70 ± 0.06 ^e^	11.90 ± 0.90 ^e^	39.67 ± 1.67 ^b^	36.40 ± 3.16 ^c^	1.40 ± 0.06 ^c^	21.93 ± 1.26 ^e^	3.50 ± 0.32 ^c^	18.67 ± 0.67 ^e^
Sids-12	175.0 ± 13.61 ^c^	449.2 ± 15.43 ^b^	303.3 ± 10.83 ^a^	379.2 ± 17.25 ^a^	301.0 ± 15.99 ^a^	330.2 ± 12.38 ^a^	193.7 ± 8.66 ^a^	324.1 ± 13.68 ^a^
Sids-13	0.70 ± 0.08 ^e^	0.70 ± 0.06 ^e^	42.23 ± 3.28 ^b^	35.43 ± 0.91 ^c^	0.70 ± 0.06 ^c^	20.07 ± 1.56 ^e^	25.67 ± 2.42 ^c^	49.00 ± 4.54 ^de^
Shandweel-1	0.00 ± 0.00 ^e^	5.13 ± 0.47 ^e^	21.47 ± 1.64 ^b^	19.60 ± 1.76 ^c^	25.43 ± 1.67 ^c^	11.43 ± 0.72 ^e^	3.50 ± 0.29 ^c^	3.50 ± 0.25 ^e^
Sakha-93	266.0 ± 27.62 ^b^	513.3 ± 23.72 ^a^	332.5 ± 16.43 ^a^	402.5 ± 18.04 ^a^	318.5 ± 17.39 ^a^	338.3 ± 18.30 ^a^	211.2 ± 18.68 ^a^	263.7 ± 14.12 ^b^
Line-35	77.43 ± 16.17 ^d^	194.1 ± 14.98 ^d^	84.47 ± 5.30 ^b^	184.3 ± 9.26 ^b^	55.77 ± 2.64 ^c^	92.17 ± 6.01 ^cd^	84.00 ± 4.54 ^b^	217.0 ± 11.52 ^c^
Line-1	0.00 ± 0.00 ^e^	10.27 ± 0.82 ^e^	0.70 ± 0.03 ^b^	19.13 ± 0.66 ^c^	0.00 ± 0.00 ^c^	104.3 ± 7.33 ^bc^	64.17 ± 6.49 ^b^	78.17 ± 3.93 ^d^
Line-2	7.93 ± 0.47 ^e^	14.00 ± 0.94 ^e^	0.00 ± 0.00 ^b^	4.20 ± 0.30 ^c^	2.33 ± 0.17 ^c^	2.33 ± 0.17 ^e^	0.00 ± 0.00 ^c^	4.67 ± 0.29 ^e^
Line-3	330.2 ± 10.37 ^a^	400.2 ± 19.27 ^c^	51.57 ± 2.33 ^b^	38.97 ± 2.47 ^c^	264.1 ± 13.96 ^b^	60.67 ± 2.74 ^d^	190.2 ± 9.92 ^a^	72.33 ± 4.28 ^d^
Line-55	0.00 ± 0.00 ^e^	0.23 ± 0.02 ^e^	0.00 ± 0.00 ^b^	4.43 ± 0.28 ^c^	0.00 ± 0.00 ^c^	0.00 ± 0.00 ^e^	0.00 ± 0.00 ^c^	0.00 ± 0.00 ^e^

E1-E8 are the evaluated environments as presented in **Table 1**. The same letters under the same environment are not significantly different by the least significant difference at p ≤ 0.05.

**Figure 1:**
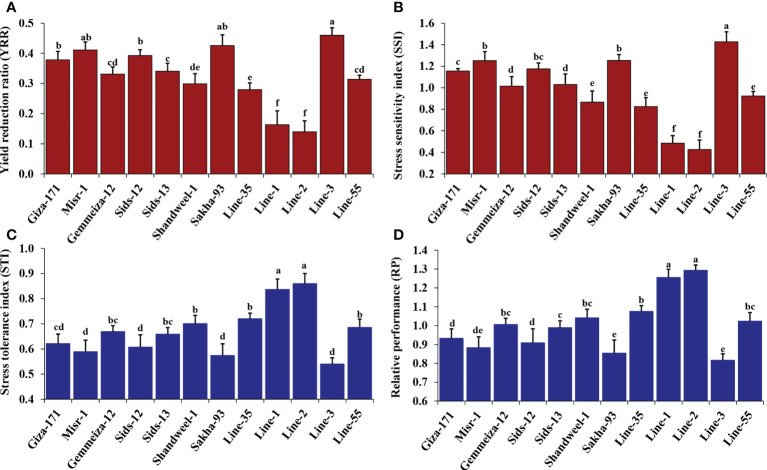
Heat tolerance indices: yield reduction ratio, YRR **(A)**, stress sensitivity index, SSI **(B)**, stress tolerance index, STI **(C)**, and relative performance, RP **(D)** for the evaluated twelve bread wheat genotypes averaged over two locations and two growing seasons. The bars on the columns represent SE, and different letters are significantly different by the least significant difference at p ≤ 0.05.

Heat stress indices were estimated for the evaluated twelve bread wheat genotypes across the tested environments (**Figures 1A-D**). The evaluated genotypes exhibited highly significant difference (P< 0.01) in all studied heat stress indices (**Table 2**). The genotypes Line-1 and Line-2 followed by Line-35, Shandweel-1 and Line-55 exhibited statistically significant (P< 0.05) lowest values of yield reduction ratio (YRR) and stress sensitivity index (SSI) (**Figures 1A**, **B**). Otherwise, Line-3 followed by Sakha-93, Misr-1, and Sids-12 possessed the highest statistically significant (P< 0.05) values for both measurements, suggesting their sensitivity to heat stress. The remaining genotypes showed moderate degrees of sensitivity to heat stress. Similarly, Line-2 and Line-1, followed by Line-35, Shandweel-1, and Line-55 recorded the highest values of stress tolerance index and relative performance (**Figures 1C**, **1D**).

### Yield traits

Highly significant differences (P< 0.01) were detected among genotypes for all studied agronomic traits. The evaluated genotypes possessed higher yield traits under timely sowing compared to late sowing (**Tables 6**-**8**). Under timely sowing, number of grains per spike varied from 44.1 (Sakha-93 at E5) to 57.0 (Line-2 at E3), while under late sowing ranged from 34.8 (Sids-13 at E4) to 51.97 (Sids-12 at E4) (**Table 6**). Likewise, 1000-grain weight varied from 31.87 (Sids-13 at E1) to 54.63 (Line-55 at E3) under timely sowing, while from 20.1 (Sakha-93 at E4) to 43.83 (Shandweel-1 at E2) (**Table 7**). Moreover, grain yield ranged from 5.29 (Line-35 under E1) to 7.83 (Line-55 under E3) ton/ha under normal sowing date (**Table 8**). Otherwise, it fluctuated from 2.6 (Line-3 under E2) to 6.01 (Line-55 under E8) ton/ha. Wheat genotypes Line-55, Gemmeiza-12, Giza-171, Line-1, Line-2, and Misr-1 were able to maintain their performance under timely and late sowing dates in all evaluated environments. Line-1, Line-2, and Line-55 produced the highest yield traits under late sowing dates in all environments.

**Table 6:** Number of grains per spike for twelve bread wheat genotypes in two locations (Kafer El-Hamam and Sakha) during two growing seasons in 2015-2016 and 2016-2017 under timely and late sowing dates.

Genotype	E1	E2	E3	E4	E5	E6	E7	E8
Giza-171	49.77 ± 1.11 ^fg^	45.70 ± 0.32 ^b^	57.23 ± 1.17 ^a^	48.80 ± 0.76 ^b^	50.87 ± 1.19 ^f^	42.87 ± 1.08 ^cd^	54.57 ± 0.86 ^abc^	47.50 ± 1.15 ^b^
Misr-1	51.23 ± 1.03 ^ef^	44.93 ± 0.61 ^bc^	53.40 ± 1.01^bc^	37.47 ± 1.07^g^	49.90 ± 0.74 ^f^	42.73 ± 1.26 ^cd^	51.20 ± 1.13 ^ef^	40.33 ± 1.14 ^ef^
Gemmeiza-12	53.73 ± 1.07 ^c^	47.63 ± 1.09 ^a^	55.03 ± 1.03 ^b^	39.97 ± 0.79 ^f^	53.40 ± 1.14 ^cd^	45.27 ± 0.64 ^ab^	52.80 ± 0.95 ^cde^	43.77 ± 1.20 ^cd^
Sids-12	49.20 ± 1.02 ^g^	43.87 ± 1.04 ^d^	55.77 ± 0.92 ^a^	51.97 ± 1.09 ^a^	57.20 ± 0.95 ^a^	44.63 ± 0.79 ^bc^	59.83 ± 0.95 ^f^	39.63 ± 1.23 ^f^
Sids-13	53.20 ± 1.14 ^cd^	44.63 ± 0.71 ^c^	56.37 ± 0.82^a^	34.80 ± 1.06 ^h^	53.43 ± 0.85 ^cd^	40.40 ± 0.86 ^e^	54.87 ± 0.72 ^ab^	36.47 ± 1.16 ^g^
Shandweel-1	52.37 ± 1.08 ^de^	45.40 ± 1.14 ^bc^	48.67 ± 1.20 ^d^	47.90 ± 1.16 ^b^	52.87 ± 1.20 ^de^	46.30 ± 1.01^ab^	56.37 ± 0.92 ^a^	45.60 ± 1.10 ^bc^
Sakha-93	45.83 ± 1.16 ^h^	41.07 ± 0.64 ^e^	51.67 ± 1.11 ^c^	45.70 ± 0.95 ^cd^	44.07 ± 1.15 ^g^	42.17 ± 1.26 ^de^	50.03 ± 1.11 ^f^	41.83 ± 0.08 ^de^
Line-35	50.13 ± 0.97 ^fg^	43.53 ± 1.13 ^d^	55.17 ± 0.72 ^ab^	37.53 ± 1.05 ^g^	53.17 ± 0.63 ^cd^	41.37 ± 1.13 ^de^	52.87 ± 1.08 ^cde^	39.80 ± 1.16 ^f^
Line-1	51.43 ± 1.01 ^ef^	45.60 ± 0.70 ^b c^	53.23 ± 1.19 ^bc^	40.83 ± 0.75 ^f^	55.07 ± 0.89 ^bc^	44.53 ± 0.87 ^bc^	53.83 ± 0.83 ^bcd^	44.40 ± 0.75 ^c^
Line-2	54.73 ± 1.09 ^b^	47.10 ± 1.02 ^a^	57.03 ± 0.76 ^a^	45.87 ± 1.03 ^c^	55.43 ± 1.17 ^ab^	46.00 ± 1.15 ^ab^	54.53 ± 0.67 ^abc^	50.80 ± 0.62 ^a^
Line-3	56.97 ± 0.83 ^a^	48.80 ± 0.85 ^a^	55.20 ± 1.16 ^ab^	43.73 ± 1.07 ^de^	51.00 ± 0.76 ^ef^	46.70 ± 0.87 ^a^	53.13 ± 0.89 ^bcd^	49.97 ± 0.72 ^a^
Line-55	54.30 ± 0.70 ^b^	45.10 ± 0.79 ^bc^	55.17 ± 1.14 ^ab^	42.93 ± 1.03 ^e^	51.70 ± 1.12^def^	42.43 ± 0.94 ^d^	52.33 ± 1.52 ^de^	44.20 ± 0.67 ^c^

E1-E8 are the evaluated environments as presented in **Table 1**. The same letters under the same environment are not significantly different by the least significant difference at p ≤ 0.05.

**Table 7:** Thousand-grain weight (g) for twelve bread wheat genotypes in two locations (Kafer El-Hamam and Sakha) during two growing seasons in 2015-2016 and 2016-2017 under timely and late sowing dates.

Genotype	E1	E2	E3	E4	E5	E6	E7	E8
Giza-171	41.27 ± 0.41 ^d^	36.30 ± 0.55 ^d^	40.20 ± 0.71 ^c^	31.50 ± 0.71 ^b^	48.10 ± 0.61 ^a^	38.03 ± 0.65 ^abc^	47.47 ± 0.88 ^a^	38.27 ± 0.92 ^bc^
Misr-1	40.20 ± 0.51 ^d^	26.60 ± 0.25 ^g^	36.80 ± 0.75 ^ef^	22.20 ± 0.85^d^	42.70 ± 0.75 ^bc^	36.77 ± 0.82 ^cd^	42.43 ± 0.75 ^d^	34.70 ± 0.59 ^ef^
Gemmeiza-12	52.37 ± 0.33 ^a^	42.40 ± 0.32 ^ab^	47.88 ± 0.88 ^b^	37.20 ± 0.68 ^a^	44.43 ± 0.67 ^b^	35.70 ± 0.53 ^de^	42.93 ± 0.84 ^cd^	34.37 ± 0.57 ^ef^
Sids-12	50.87 ± 0.47 ^a^	30.87 ± 0.35 ^f^	48.87 ± 0.45 ^b^	25.83 ± 0.82 ^c^	40.43 ± 0.71 ^d^	34.17 ± 0.66 ^ef^	40.97 ± 0.79 ^def^	33.63 ± 0.44 ^fg^
Sids-13	31.87 ± 0.38^g^	26.50 ± 0.23^g^	37.57 ± 0.53^def^	21.47 ± 0.81 ^de^	39.63 ± 0.64 ^d^	29.93 ± 0.82 ^hi^	35.37 ± 0.86 ^g^	27.07 ± 0.84 ^i^
Shandweel-1	35.50 ± 0.25^f^	43.83 ± 0.68^a^	40.67 ± 0.32 ^c^	35.70 ± 0.56 ^a^	40.90 ± 0.86 ^cd^	36.10 ± 0.76 ^cde^	40.87 ± 0.79 ^ef^	35.67 ± 0.78 ^de^
Sakha-93	38.17 ± 0.23 ^e^	26.43 ± 0.33^g^	33.90 ± 0.54^g^	20.10 ± 0.87 ^e^	39.77 ± 0.77 ^d^	28.43 ± 0.49 ^i^	33.07 ± 0.76 ^h^	28.70 ± 0.59 ^i^
Line-35	43.77 ± 0.52 ^c^	39.07 ± 0.19^c^	35.87 ± 0.52 ^fg^	30.57 ± 0.79 ^b^	44.33 ± 0.56 ^b^	33.47 ± 0.83^fg^	44.67 ± 0.81 ^bc^	31.77 ± 0.62^gh^
Line-1	37.70 ± 0.32 ^e^	34.00 ± 0.45^e^	38.53 ± 0.61 ^de^	27.70 ± 0.98 ^c^	39.77 ± 0.59 ^d^	31.60 ± 0.82 ^gh^	39.03 ± 0.75 ^f^	31.30 ± 0.67 ^h^
Line-2	40.90 ± 0.56 ^d^	35.73 ± 0.46 ^de^	37.03 ± 0.37 ^def^	31.30 ± 0.75 ^b^	44.27 ± 0.84 ^b^	37.80 ± 0.72^bc^	46.10 ± 0.81 ^ab^	37.00 ± 0.72 ^cd^
Line-3	41.80 ± 0.41 ^cd^	40.63 ± 0.23 ^bc^	39.00 ± 0.49 ^cd^	37.40 ± 0.59 ^a^	41.33 ± 0.71 ^cd^	39.90 ± 0.44 ^a^	41.73 ± 0.84 ^de^	39.03 ± 0.64 ^b^
Line-55	48.57 ± 0.22 ^b^	42.37 ± 0.59^ab^	54.63 ± 0.76 ^a^	37.00 ± 0.46 ^a^	47.43 ± 0.87 ^a^	39.13 ± 1.67 ^ab^	46.37 ± 0.75 ^ab^	41.57 ± 0.79 ^a^

E1-E8 are the evaluated environments as presented in **Table 1**. The same letters under the same environment are not significantly different by the least significant difference at p ≤ 0.05.

**Table 8:** Grain yield (t/ha) for twelve bread wheat genotypes in two locations (Kafer El-Hamam and Sakha) during two growing seasons in 2015-2016 and 2016-2017 under timely and late sowing dates.

Genotype	E1	E2	E3	E4	E5	E6	E7	E8
Giza-171	6.83 ± 0.11 ^ab^	3.93 ± 0.31 ^c^	6.94 ± 0.36 ^ab^	4.62 ± 0.18 ^abcd^	7.46 ± 0.21^a^	4.12 ± 0.29 ^b^	7.58 ± 0.23 ^a^	4.86 ± 0.14 ^bc^
Misr-1	6.59 ± 0.29 ^abc^	4.48 ± 0.15 ^bc^	7.13 ± 0.31 ^a^	4.35 ± 0.28 ^bcd^	7.53 ± 0.38 ^a^	3.49 ± 0.34 ^b^	7.13 ± 0.25^ab^	5.12 ± 0.27 ^abc^
Gemmeiza-12	6.97 ± 0.16 ^a^	4.45 ± 0.18 ^bc^	6.68 ± 0.43 ^bc^	4.74 ± 0.31^abc^	6.47 ± 0.21 ^b^	4.23 ± 0.18 ^b^	6.78 ± 0.24 ^abc^	4.55 ± 0.32 ^c^
Sids-12	5.45 ± 0.27 ^ef^	4.07 ± 0.26 ^bc^	6.33 ± 0.34 ^bcd^	3.36 ± 0.26 ^e^	6.21 ± 0.27 ^bc^	3.96 ± 0.21^b^	6.37 ± 0.32 ^bcd^	3.22 ± 0.19 ^d^
Sids-13	6.42 ± 0.26 ^abcd^	4.76 ± 0.26 ^abc^	5.95 ± 0.22^cd^	3.86 ± 0.24 ^cde^	5.38 ± 0.31 ^c^	3.90 ± 0.35^b^	5.47 ± 0.30 ^e^	2.80 ± 0.12 ^d^
Shandweel-1	5.83 ± 0.17 ^cdef^	4.81 ± 0.30^abc^	6.62 ± 0.38 ^bc^	5.16 ± 0.28 ^ab^	6.77 ± 0.30^ab^	2.88 ± 0.21 ^c^	6.44 ± 0.28 ^bcd^	4.88 ± 0.29 ^bc^
Sakha-93	5.55 ± 0.24 ^def^	4.17 ± 0.15^bc^	6.38 ± 0.28^bcd^	3.12 ± 0.18 ^e^	6.08 ± 0.23 ^bc^	3.18 ± 0.19 ^c^	5.68 ± 0.31 ^de^	2.81 ± 0.36 ^d^
Line-35	5.29 ± 0.14 ^f^	4.39 ± 0.20 ^bc^	5.36 ± 0.30 ^e^	3.90 ± 0.34 ^cde^	6.00 ± 0.32 ^bc^	3.66 ± 0.25 ^b^	6.95 ± 0.28 ^abc^	4.89 ± 0.17 ^bc^
Line-1	6.04 ± 0.24 ^bcdef^	5.54 ± 0.31^a^	5.97 ± 0.23 ^cde^	5.25 ± 0.23 ^a^	6.40 ± 0.35 ^b^	5.59 ± 0.16 ^a^	6.20 ± 0.19 ^cde^	5.58 ± 0.18 ^ab^
Line-2	6.39 ± 0.21 ^abcde^	5.56 ± 0.26 ^a^	5.63 ± 0.25 ^de^	5.06 ± 0.54 ^ab^	6.14 ± 0.16 ^bc^	5.47 ± 0.29 ^a^	6.72 ± 0.32 ^abc^	5.20 ± 0.30 ^abc^
Line-3	5.97 ± 0.25 ^bcdef^	2.68 ± 0.13^d^	5.89 ± 0.32 ^cde^	3.25 ± 0.18 ^e^	6.81 ± 0.24 ^ab^	3.92 ± 0.25 ^b^	6.06 ± 0.34 ^cde^	3.51 ± 0.17 ^d^
Line-55	5.74 ± 0.30^cdef^	4.83 ± 0.35 ^ab^	7.83 ± 0.26 ^a^	3.79 ± 0.19^de^	6.05 ± 0.26 ^bc^	3.69 ± 0.31 ^b^	7.36 ± 0.37 ^a^	6.01 ± 0.13 ^a^

E1-E8 are the evaluated environments as presented in **Table 1**. The same letters under the same environment are not significantly different by the least significant difference at p ≤ 0.05.

### Genotypic classification based on rust resistance, heat tolerance, and yield traits

**Figure 2:**
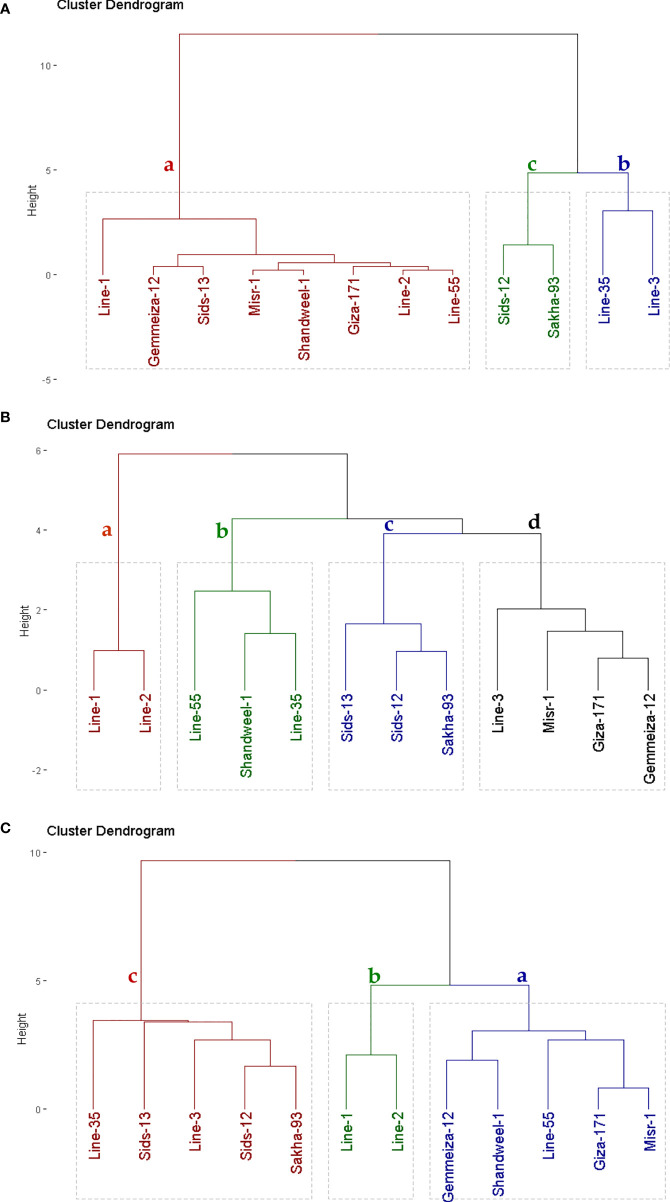
Dendrogram of the phenotypic distances among twelve bread wheat genotypes based on the yellow rust measurements **(A)**, heat tolerance indices **(B)**, and yield traits **(C)** in eight environments.

Yellow rust measurements, heat tolerance indices, and yield traits were utilized to categorize the evaluated genotypes into different groups. Using hierarchical clustering the genotypes were grouped into three clusters according to yellow rust measurements (**Figure 2A**). Group (a) included eight genotypes that had the lowest values of severity percentage, the average coefficient of infection (ACI), and the area under disease progress curve (AUDPC). Accordingly, this group could be considered resistant genotypes. Groups (b) and (c) contained two genotypes that presented intermediate and low levels of rust resistance. Heat tolerance indices; stress tolerance index (STI), and relative performance (RP) were utilized to differentiate the evaluated genotypes into tolerant and sensitive genotypes (**Figure 2B**). Four groups were identified by employing hierarchical clustering. Group (a) comprised two genotypes that had the highest values of tolerance indices (STI and RP). Group (b) and (c) contained three genotypes with intermediate values while group (d) had four genotypes with the lowest values of tolerance indices (STI and RP). Yield traits; number of grains/spike, 1000-grain weight, and grain yield were employed to classify the evaluated genotypes based on their agronomic performance into three groups (**Figure 2C**). Five genotypes in group (a) possessed the highest yield traits, group (b) consisted of two genotypes with intermediate yield traits, and group (c) with five genotypes produced the lowest yield traits (**Figure 2C**).

### Grain yield stability

**Figure 3:**
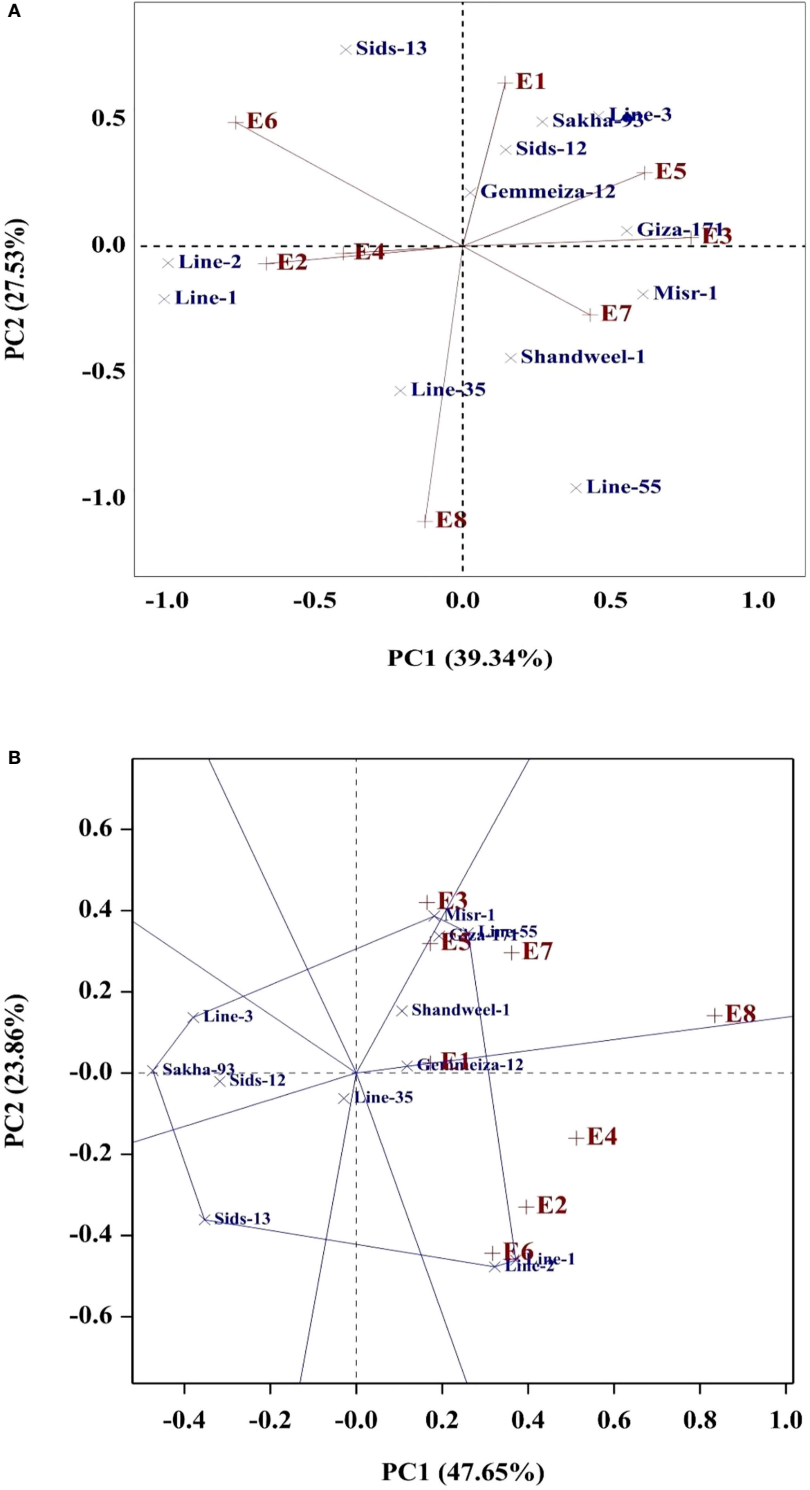
AMMI **(A)** and GGE **(B)** biplots for grain yield of the evaluated twelve bread wheat genotypes in eight environments (E1-E8) which are explained in **Table 1**.

Several statistical procedures are employed to explore the state of environmental effect on the evaluated genotypes. In the present study, regression slope (b_i_), deviation from linear regression (S^2^d_i_), stratified ranking (TOP), Wricke’s Ecovalence (WE), cultivar superiority (CS), and AMMI Stability value (ASV) were applied to assess genotype stability due to their simplicity. Additionally, the models of AMMI and GGE were performed as they are effective analytical procedures to identify the genotype by environment interaction graphically. The phenotypic stability implied that the regression coefficient (b_i_) for grain yield of twelve bread wheat genotypes fluctuated from 0.78 (Line-35) to 1.25 (Misr-1), reflecting the genetic variations among the evaluated genotypes in their regression response for grain yield (**Table 9**). The b_i_ values were diverged considerably more than the unity (b_i_>1) in genotypes Giza-171, Misr-1, Sakha-93, and Line-3, indicating they relatively suitable for favorable environments. Otherwise, the b_i_ values deviated considerably and were less than unity (b_i_<1) in Sids-13, Shandweel-1, Line-35, and Line-2. Subsequently, these genotypes could be adapted to unfavorable environments. Furthermore, Giza-171, Gemmeiza-12, Sids-12, Misr-1, and Line-2 displayed lower deviations from regression (S^2^d_i_) for grain yield. Giza-171, Misr-1, Gemmeiza-12, Line-1, Line-2, and Line-55 exhibited the highest values of stratified ranking (TOP) and had the best performance across the tested environments. Gemmeiza-12, Sids-12, Shandweel-1, Sakha-93, and Line-35 exhibited the lowest ASV as the most desired and stable genotypes. Otherwise, the other genotypes were more responsive and exhibited higher values of ASV. Likewise, Giza-171, Misr-1, Gemmeiza-12, Sids-12, Sakha-93, and Line-35 displayed the lowest values of Wricke’s ecovalence (WE). Besides, the lowest values of cultivar superiority (CS) were assigned for Giza-171, Misr-1, Gemmeiza-12, Line-1, Line-2, and Line-55. The PC1 divided the environments into groups, with timely sowing on the positive side, and late sowing on the negative side of PC1 (**Figure 3A**). The tested environments contributed with different magnitude in grain yield variation. The environments E6 and E8 were the most differentiating environments and showed considerable contribution to G×E and were located far away from the origin (**Figures 3A**, **B**). The evaluated genotypes exhibited different PCs scores and accordingly different G×E performance. Gemmeiza-12, Sids-12, Giza-171, Misr-1, Sakha-93, Shandweel-1, Line-3, and Line-55 exhibited specific adaptation for timely sowing (E1, E3, E5 and E7). Otherwise, more adapted genotypes with late sowing were Line-1 and Line-2. Gemmeiza-12, Sids-12, Shandweel-1, Sakha-93, and Line-35 displayed the least PCs values, and accordingly lower G×E interaction and more stable (**Figures 3A**, **B**).

**Table 9:** Stability parameters of twelve bread wheat genotypes tested in eight environments comprising of two locations (Kafer El-Hamam and Sakha), two growing seasons (2015-2016 and 2016-2017) and two sowing dates (timely and late sowing).

Genotype	GY	b_i_	${\text{S}}_{d}^{2}$	TOP	ASV	WE	CS
Giza-171	5.79	1.15	1.29	71.88	0.81	1.83	0.46
Misr-1	5.73	1.25	1.89	71.88	0.91	1.76	0.50
Gemmeiza-12	5.61	0.92	1.03	69.79	0.22	0.62	0.54
Sids-12	4.87	1.03	1.37	37.50	0.44	1.24	1.50
Sids-13	4.82	0.79	4.58	36.46	0.98	3.50	1.79
Shandweel-1	5.42	0.89	3.34	58.33	0.51	2.64	0.87
Sakha-93	4.62	1.18	2.28	26.04	0.64	1.87	2.02
Line-35	5.06	0.78	2.09	37.50	0.66	1.88	1.24
Line-1	5.82	0.92	2.11	71.88	1.49	4.35	0.48
Line-2	5.77	0.88	1.09	67.71	1.46	3.85	0.54
Line-3	4.76	1.21	3.85	37.50	0.85	3.11	1.81
Line-55	5.66	1.01	6.61	63.54	1.13	4.92	0.63

GY, grain yield (t/ha); b_i_, regression slop; ${\text{S}}_{d}^{2}$ , deviation from linear regression; TOP, the stratified ranking; ASV, AMMI stability value; WE, Wricke’s Ecovalence values; CS, Cultivar superiority.

### Interrelationships among evaluated genotypes and measured traits

**Figure 4:**
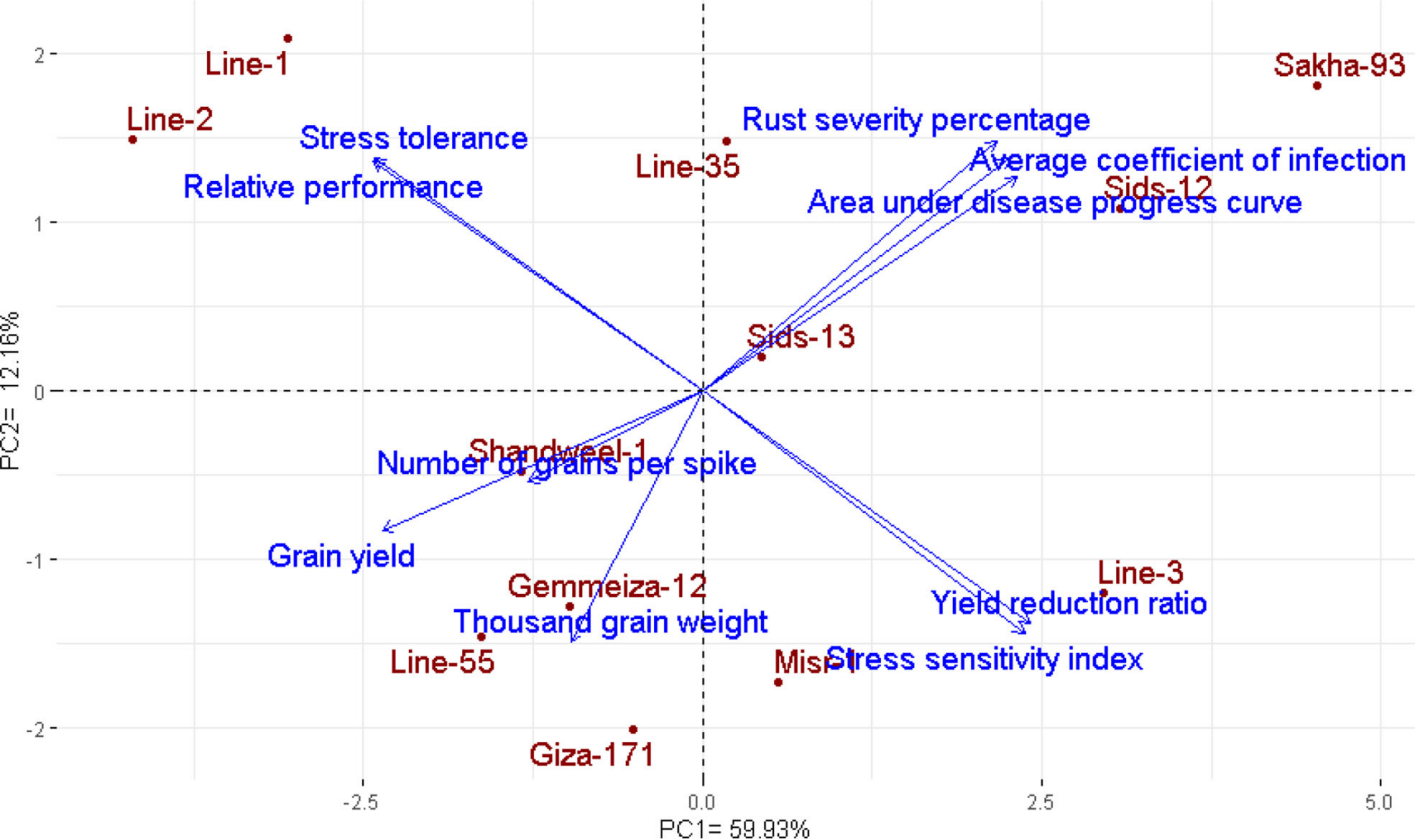
Biplot of PCA for the evaluated twelve bread wheat genotypes based on yellow rust resistance measurements, heat tolerance indices, and agronomic performance.

**Figure 5:**
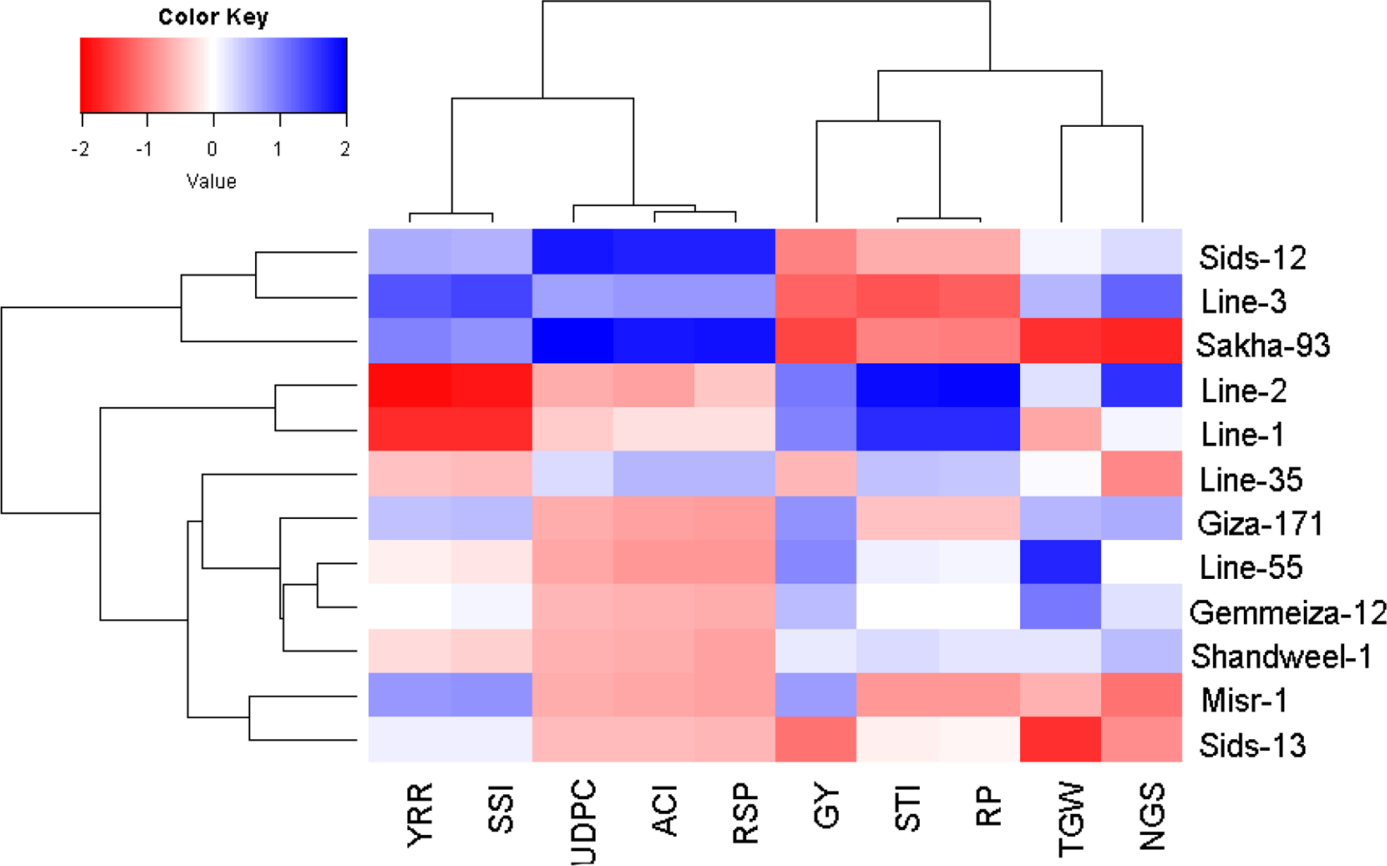
Heatmap and hierarchical clustering divide the evaluated twelve bread wheat genotypes into different clusters based on yellow rust resistance measurements, heat tolerance indices, and agronomic performance. Red and blue colors imply low and high values for the corresponding parameters, respectively. RSP, rust severity percentage, ACI, the average coefficient of infection, AUDPC, area under disease progress curve, YRR, yield reduction ratio, SSI, stress sensitivity index, STI, stress tolerance index, RP, relative performance, NGS, number of grains per spike, TGW, 1000-grain weight, and GY, grain yield.

The multivariate analysis implied that the first two PCAs explained 72.09% of the total variation (**Figure 4**). The evaluated parameters were divided into four groups, yellow rust measurements, yield traits, and two groups for heat tolerance indices. Sakha-93 and Sids-12 were associated with high values of yellow rust measurements and were situated oppositely to yield traits. Otherwise, Line-55, Shandweel-1, Gemmeiza-12, and Giza-171 were positively associated with yield traits and had a negative relationship with the rust measurement values. Likewise, Line-1 and Line-2 were positively associated with heat tolerance indices STI and RP while negatively correlated with YRR and SSI which were associated with Line-3 and Misr-1. Correspondingly, the heatmap and hierarchical clustering based on the recorded parameters; yellow rust measurements, heat tolerance indices, and yield traits, divided the evaluated genotypes into three main clusters (**Figure 5**). Line-1 and Line-2 had the best performance for all evaluated parameters with high yield traits, STI and RP, and low rust measurements, SSI, and YRR. Followed by the second cluster containing Giza-171, Line-55, Line-35, Gemmeiza-12, Shandweel-1, Misr-1, and Sids-13. Otherwise, Sids-12, Line-3, and Sakha-93 exhibited lower yield traits, STI and RP, and higher rust measurements, SSI and YRR.

## Discussion

Yellow rust and heat stress adversatively impact the productivity of bread wheat in particular under increasing adverse environmental conditions and abrupt climate change (Latif et al., 2020; Martínez-Moreno et al., 2022). Several previous studies explored rust resistance, heat tolerance, agronomic performance, and yield adaptability of wheat genotypes severally. But in the present study the rust resistance, heat resilience, and yield potentiality, as well as yield stability were assessed simultaneously in different locations during two growing seasons under two sowing dates employing several statistical procedures.The obtained results exhibited highly significant variations among the evaluated genotypes in all studied measurements signifying the presence of an adequate degree of genetic variability for yellow rust resistance, heat stress, and yield traits in the used plant material. Likewise, the evaluated environments including locations and sowing dates displayed significant differences indicating their significant impacts on yellow rust resistance, heat stress, and yield traits.

The evaluated genotypes displayed a wide range of yellow rust resistance. The results indicated that Giza-171, Misr-1, Gemmeiza-12, Sids-13, Shandweel-1, Line-1, Line-2, and Line-55 provided lower values of FRS, ACI, and AUDPC and accordingly were marked to be superior in rust resistance under all evaluated environments compared to the other genotypes. The most prevalent observation is the genotype Line-55 which recorded the lowest values of all estimated yellow rust parameters in all evaluated environments. Otherwise, Sids-12, Sakha-93, Line-3, and Line-35 displayed high values of FRS, ACI, and AUDPC and were obvious to have lower levels of rust resistance and were susceptible to yellow rust infection. In this context, (Naseri and Marefat, 2019) elucidated highly significant differences in AUDPC among evaluated eight commercial wheat cultivars under four sowing dates. Likewise, Elkot et al. (2016) recorded highly genetic differences between local wheat cultivars and some exotic genotypes in the genes controlling yellow rust resistance. Moreover, Boulot and Aly (2014) tested local wheat cultivars and detected only four cultivars that have an adequate level of partial resistance and possessed lower values of FRS and AUDPC. On the contrary, three highly susceptible ones with higher values of FRS and AUDPC. Furthermore, Ali et al. (2018) assessed yellow rust resistance among twenty wheat genotypes. Their results manifested that the evaluated genotypes possessed considerable diversity concerning slow rusting behavior field resistance varying from immunity to partial resistance. The genotypes were grouped into different clusters based on their resistance to the yellow rust. Nine resistant genotypes were grouped in one cluster, while the remaining eleven genotypes were gathered in another group. Additionally, Mabrouk et al. (2022) tested yellow rust in different wheat genotypes. They employed the disease parameters *i.e*., FRS and AUDPC to differentiate the resistant and susceptible genotypes.Their results displayed substantial differences among the evaluated genotypes in FRS and AUDPC scores. Two wheat genotypes recorded the lowest values of the disease parameters during the two seasons compared to the check susceptible variety Morocco and the other evaluated genotypes. Besides, the two growing seasons differed in the disease measurements with higher values in the first season than the second one.

The environmental factors displayed significant impacts on yellow rust resistance. The estimates of yellow rust measurements varied from one environment to another. Generally, late sowing displayed higher FRS, ACI and AUDPC compared to timely sowing. In this respect, Chen et al. (2014) and (Naseri and Kazemi, 2020) depicted that late sowing increases rust infection in wheat by providing favorable air temperature and relative humidity that are suitable for infection. Otherwise, early sowing has decisive importance in weakening rust severity (Singh et al., 2012; Atiq et al., 2017). Considerable consistency was detected between the two tested sowing dates for Giza-171, Misr-1, Gemmeiza-12, Sids-12, Sids-13, and Line-55. Otherwise, the impact of late sowing was more pronounced in the susceptible genotypes Sids-12, Sakha-93, Line-3 and Line-35.

Heat stress usually peaks at postanthesis and throughout the grain filling period (Schittenhelm et al., 2020). The morphological and physiochemical changes due to heat stress during the postanthesis stage reduce grain yield (Marcos-Barbero et al., 2021). The performances of highly-yielding genotypes are weakly predictable across shifted sowing dates due to heat stress at this stage. The genotypes with improved yield traits should be evaluated for their stability in withstanding erratic temperature situations at different locations and diverse sowing dates (Dias and Lidon, 2009). Plant breeders frequently perform trials to identify stable genotypes that are capable of withstanding postanthesis stresses. Heat-resilient genotypes lessen the adverse impacts of high temperature at the postanthesis phase and maintain high grain yield (Fernie et al., 2022). In the present study, the evaluated wheat genotypes were assessed under late sowing to expose the plants to heat stress at the anthesis and grain-filling period. Different indices for heat stress tolerance were employed to discriminate the tolerant genotypes and sensitive ones. The yield reduction ratio (YRR), stress sensitivity index (SSI), relative performance (RP), and stress tolerance index (STI) displayed that Line-1 and Line-2 followed by Line-35, Shandweel-1, and Line-55 are more tolerant to heat stress compared to the remaining genotypes. Otherwise, Line-3, Sakha-93, Misr-1, and Sids-12 exhibited the highest values of YRR and SSI while the lowest values of STI and RP suggest their sensitivity to high-temperature circumstances. Similarly, Kamara et al. (2021) evaluated diverse wheat genotypes in two locations under timely and late sowing conditions. They employed stress tolerance index and yield index to classify the assessed genotypes according to their heat stress tolerance. Moreover, Kamrani et al. (2018) evaluated diverse genotypes under normal sowing and late sowing conditions to assess their heat tolerance. They used stress tolerance index, geometric mean productivity, and mean productivity indices to identify heat tolerant and high-yielding genotypes. Furthermore, Youldash et al. (2020) assessed heat tolerance of diverse wheat genotypes by evaluating under optimal conditions and late sowing. They demonstrated that the heat stress sensitivity index is efficient to distinguish the heat tolerant and sensitive genotypes. Generally, the variation in yield traits among different environments is due to the alteration in the environmental elements and their interaction with the genotypes (Yan and Tinker, 2005). In the present study, the environmental effect displayed the highest proportion of total variation of yield traits followed by genotype by environment and genotypic effects. The highly significant effect of the environmental component as well as G×E demonstrates that the grain yield was highly affected by the studied combination of environmental aspects; seasons, locations, and sowing dates. The environmental variation in yield traits was obviously dominated by the sowing date effect due to exploring wheat plants to unfavorable conditions and environmental stress. Yield traits under timely sowing were higher than under late sowing. This is due to the suitability of the environmental circumstances for the growth and development of wheat at the optimum time compared to delaying the sowing date. Furthermore, detected highly significant genotype by environment interaction for yield traits disclosing that wheat genotypes contrasted in their response to the environmental variations (Gracia et al., 2012; Mansour et al., 2020; Kamara et al., 2022). The genotypes Line-55, Gemmeiza-12, Giza-171, Line-1, Line-2, and Misr-1 were able to maintain acceptable agronomic performance under timely and late sowing dates in all evaluated environments. Indeed, Line-1, Line-2, and Line-55 produced the highest yield traits under late sowing dates in all environments.

Assessing the stability of grain yield across different environments is essential for enhancing wheat production (Macholdt and Honermeier, 2017). In the present study, different statistical procedures were employed to explore the adaptability and stability of tested genotypes *i.e*., joint regression, stratified ranking, Wricke’s Ecovalence values, cultivar superiority, additive main effects, and multiplicative interaction (AMMI), AMMI stability value, and genotype plus genotype-by-environment interaction (GGE). The applied stability parameters were quite similar for describing the stability of the evaluated wheat genotypes. Simultaneous consideration of the applied stability parameters, Gemmeiza-12, Giza-171, Sids-12, Sids-13, Misr-1 Shandweel-1, Line-1, Line-2, and Line-55 possessed desirable and stable performance across the evaluated environments. This provides evidence of utilizing these genotypes in breeding programs for enhancing wheat grain yield stability and productivity principally under abrupt climate change and increasing adverse environmental conditions. Similarly, significant variations in the stability of wheat grain yield and specific adaptation for certain genotypes to specific environments were reported by Roostaei et al. (2014); Kapoor et al. (2020); Alipour et al. (2021); Awaad (2021); Omrani et al. (2022)

## Conclusions

The evaluated genotypes exhibited highly significant differences in yellow rust resistance, heat stress tolerance, and yield traits demonstrating the presence of an adequate degree of genetic variability in the used plant material. The genotypes Giza-171, Misr-1, Gemmeiza-12, Sids-13, Shandweel-1, Line-1, Line-2, and Line-55 had better yellow rust resistance. Otherwise, Sids-12, Sakha-93, Line-3, and Line-35 displayed low levels of rust resistance to yellow rust infection. Line-1, Line-2 Line-35, Shandweel-1, and Line-55 were classified as more tolerant to heat stress compared to the remaining genotypes. On the contrary, Line-3, Sakha-93, Misr-1, and Sids-12 were less tolerant to high-temperature circumstances and could be considered sensitive ones. The genotypes Line-55, Gemmeiza-12, Giza-171, Line-1, Line-2, and Misr-1 were able to maintain acceptable agronomic performance under timely and late sowing dates in all evaluated environments and were desirable stable. In general, the genotypes Line-1 and Line-2 had the best performance for rust resistance, heat tolerance, and agronomic performance followed by Giza-171, Line-55, Line-35, Gemmeiza-12, Shandweel-1, Misr-1, and Sids-13. The detected promising genotypes for rust resistance, heat tolerance, and agronomic performance will be exploited in the wheat breeding program for improving its productivity and stability mainly under the current climate change.

## Data availability statement

The raw data supporting the conclusions of this article will be made available by the authors, without undue reservation.

## Author contributions

EMAM, HA, IR, MA-H and AS conceived and designed the experiments. DE-N and EM helped in conducting the experiments and collected the literature. MA-H and EM analyzed the data and drafted the manuscript. EMAM, HA, MA-H and EM wrote and made the final edits to the manuscript. All the authors read and approved the final version of the manuscript. All authors contributed to the article and approved the submitted version.

## Conflict of interest

The authors declare that the research was conducted in the absence of any commercial or financial relationships that could be construed as a potential conflict of interest.

## Publisher’s note

All claims expressed in this article are solely those of the authors and do not necessarily represent those of their affiliated organizations, or those of the publisher, the editors and the reviewers. Any product that may be evaluated in this article, or claim that may be made by its manufacturer, is not guaranteed or endorsed by the publisher.
