# Supplementary material for: Assessing performance and stability of yellow rust resistance, heat tolerance, and agronomic performance in diverse bread wheat genotypes for enhancing resilience to climate change under Egyptian conditions
Source: Front Plant Sci. 2022 Nov 10;13:1014824. doi: 10.3389/fpls.2022.1014824 (PMC9686012; doi:10.3389/fpls.2022.1014824)
Supplement: Supplementary file 1 [file DataSheet_1.docx]

**Table S1.** Pedigree and origin of the evaluated wheat genotypes

| **Origin** | **Pedigree** | **Genotype** |
| --- | --- | --- |
| EGYPT | OASIS/KAUZ//4*BCN/3/2*PASTOP  CMss00Y01881T-050M-030Y-030M-030WGY-33M-0Y-0S | Misr-1 |
| EGYPT | BUC//7C/ALD/5/MAYA74/ON//1160.47/3/BB/GLL/4/CHAT"S"/6/MAYA/VUL//CMH74A.630/4/*5X. | Sids-12 |
| EGYPT | SAKHA92/TR810328. | Sakha-93 |
| EGYPT | SITE/MO/4/NAC/TH.AC//3*PVA/3/MIRLO/BUC. | Shandweel 1 |
| EGYPT | SAKHA 93 / GEMMIEZA 9 S.6-1GZ-4GZ-1GZ-2GZ-0S  N.S.732/Pim/Vee"S" | Giza 171 |
| EGYPT | Kauz‘‘s‘‘//Tsi / Snb‘‘s ‘‘ | Sids 13 |
| EGYPT | OTUS/3/SARA/THB//VEE.CCMSS97Y00227S-5Y-010M-010Y-010M-2Y-1M-0Y-0GM | Gemmeiza12 |
| Mexico | KS82W418/SBN/3/CHEN/AE.SQ//2*OPATA/4/FRET2. | Line 35 |
| Sakha promising  Line #10 | CMH80A.768/3*CNO79/4/CS/TH.SC//3*PVN/3/MIRLO/BUC/5/PRINIA/6/SSERII/MILAN | Line-1 |
| SakhaPromissing #2 | GEMMEIZA10/WBLLI*2/BRAMBLING | Line-2 |
| SakhaPromissing#7 | GEMMEIZA#3/7/VEE/CMH77A917//VEE/3/GANFRENCH/6/CMH79 | Line-3 |
| NBWSN014/015 83 | SW89.5181/KAUZ/4/MILAN/KAUZ//PRINIA/3/BABAX  ACS-W-10550-013S-015S-10S-0S | Line 55 |

**Table S2.** Monthly weather data during the field trial at Kafer El-Hamam, Sharkia and Sakha, Kafer El- Shekh Governorates during the two growing seasons 2015 -2016 and 2016-2017

| **Month** | **2015-2016** | | | | **2016-2017** | | | |
| --- | --- | --- | --- | --- | --- | --- | --- | --- |
|  | Rain (mm) | RH (%) | Tmax  (°C) | Tmin  (°C) | Rain (mm) | RH (%) | Tmax  (°C) | Tmin  (°C) |
| **Kafer El-Hamam** | | | | |  | | | |
| November | 0.64 | 67.99 | 23.44 | 20.31 | 0.78 | 63.55 | 23.61 | 20.15 |
| December | 0.27 | 67.58 | 19.19 | 16.17 | 1.11 | 68.11 | 18.05 | 14.78 |
| January | 0.58 | 67.15 | 16.78 | 12.96 | 0.09 | 67.15 | 16.30 | 12.66 |
| February | 0.09 | 71.18 | 19.23 | 14.62 | 0.21 | 71.18 | 16.84 | 13.41 |
| March | 0.24 | 68.17 | 20.28 | 15.31 | 0.01 | 68.17 | 19.37 | 14.94 |
| April | 0.08 | 68.29 | 24.10 | 18.31 | 1.38 | 68.29 | 21.56 | 16.66 |
| **Sakha** | | | | |  | | | |
| November | 8.05 | 67.11 | 26.18 | 16.07 | 0.12 | 59.83 | 27.03 | 15.39 |
| December | 2.00 | 67.82 | 21.40 | 11.66 | 2.54 | 70.02 | 19.29 | 9.52 |
| January | 3.46 | 66.53 | 18.73 | 8.30 | 0.71 | 70.55 | 18.31 | 6.95 |
| February | 0.42 | 62.57 | 24.45 | 9.94 | 1.55 | 68.04 | 20.85 | 8.08 |
| March | 0.80 | 56.64 | 26.38 | 11.56 | 1.28 | 60.34 | 24.98 | 10.87 |
| April | 0.51 | 50.13 | 33.39 | 14.83 | 1.10 | 58.08 | 28.71 | 12.55 |

Rain is precipitation (mm day^-1^), RH is average relative humidity at 2 meters,Tmaxis average maximum temperature, Tminis average minimum temperature, Tmeanis temperature average.

**Table 3.** Soil mechanical and chemical analysis of the experimental sites.

| PH | Available K  (ppm) | Available P  (ppm) | Available N  (ppm) | Texture  class | Clay  (%) | Silt  (%) | Sand  (%) | Year | Site |
| --- | --- | --- | --- | --- | --- | --- | --- | --- | --- |
| 8.31 | 271.6 | 19.8 | 41.1 | Clay | 46.8 | 34.5 | 18.7 | 16-2015 | Kafer El Hamam |
| 8.22 | 300.4 | 18.4 | 32.2 | Clay | 48.9 | 33.6 | 17.5 | 2016-17 |  |
| 7.89 | 180.7 | 23.1 | 24.4 | Clay | 50.8 | 29.8 | 19.4 | 16-2015 | Sakha |
| 8.12 | 190.6 | 22.6 | 30.2 | Clay | 52.3 | 28.9 | 18.8 | 2016-17 |  |
